# Supplementary material for: Content-rich biological network constructed by mining PubMed abstracts
Source: BMC Bioinformatics. 2004 Oct 8;5:147. doi: 10.1186/1471-2105-5-147 (PMC528731; doi:10.1186/1471-2105-5-147)
Supplement: Additional File 2 — The original results of the above study (non-essential files are deleted to keep the file size under the limit set by BMC bioinformatics). [file 1471-2105-5-147-S2.bz2 › chilibotAdditionalFile2/dip20/426ID11470772E10356/html/CCR1_CCL1.html]

 


 **CCR1** and **CCL1** 
  
Found 34 abstracts in PubMed, retrieved 20.  
 

 What does Google say? 
 PDF only 
| .edu only 

---

**Interactive relationship** (e.g. stimulation, inhibition, etc)

**Neutral relationship**- Natural proteolytic processing of hemofiltrate CC chemokine 1  [ **CCL1** ]  generates a potent CC chemokine receptor CCR 1  [ **CCR1** ]  and CCR5 agonist with anti HIV properties.  Ref: 11085751 J Exp Med, 2000
- Proteolytic processing of the abundant plasmatic human CC chemokine 1  [ **CCL1** ]  HCC 1 generates a truncated form, HCC 1 9 74, which is a potent agonist of  **CCR1** , CCR3, and CCR5.  Ref: 11897579 Antimicrob Agents Chemother, 2002

**Non-interactive relationship** (e.g. studied together, co-existance, homology, etc.)

- Renal function urine volume UV, 24 h excretion of Na, K and Cl UNA V, UK V, UCl V, clearance of Na CNa, K CK and Cl CCl  [ **CCL1** ] , creatinine CCr  [ **CCR1** ] , osmotic substances Cosm, and solute free water FWC, and the fractional excretion of Na, K and Cl and ALDO were measured for an additional 3 consecutive days during the training period.  Ref: 12405745 Equine Vet J Suppl, 2002
- Nevertheless, intermediate maturity DCs expressed substantial levels of Flt 3L, IGF 1, IL 1alpha and 1beta, IL 6, CCL  [ **CCL1** ] , 1 7, and 22, MIP 2, osteopontin, CCR  [ **CCR1** ] , 5, and 7, and CXCR 4.  Ref: 11902830 Cell Immunol, 2001
- The detection of the CC chemokine receptor CCR  [ **CCR1** ]  8 message in human umbilical vein endothelial cells HUVECs by reverse transcription polymerase chain reaction RT PCR and RNase protection assay RPA, prompted us to investigate the potential role exerted by the CC chemokine I 309  [ **CCL1** ] , a known ligand of such receptor, in both in vitro and in vivo angiogenesis assays.  Ref: 11110671 Blood, 2000
- To test the in vivo relevance of this, we used a murine melanoma system and knockout mice to investigate the function of the chemokine receptor CCR5 and its ligands, CCR  [ **CCR1** ]  ligand CCL  [ **CCL1** ]  3 and CCL5.  Ref: 12682253 J Immunol, 2003
- The most active chemokines for resting Th IL 10, in terms of calcium mobilization and in vitro migration, were in order of potency CCL2 monocyte chemoattractant protein 1, CCR2 ligand, CCL4 macrophage inflammatory protein 1beta, CCR5 ligand, CCL3 macrophage inflammatory protein 1alpha,  **CCR1**  5 ligand, CCL17 thymus and activation regulated chemokine, CCR4 ligand,  **CCL1**  I 309  [ **CCL1** ] , CCR8 ligand, CXCL12 stromal derived factor 1, CXCR4, and CCL11 eotaxin, CCR3 ligand.  Ref: 11145678 J Immunol, 2001
- We have previously isolated from human hemofiltrate an N terminally truncated form of the hemofiltrate CC chemokine 1  [ **CCL1** ]  HCC 1, and characterized HCC 1 9 74 as a strong agonist of  **CCR1** , CCR5, and to a lower extent CCR3.  Ref: 11544332 J Immunol, 2001
- CCL  [ **CCL1** ]  receptors, CCR2,  **CCR1** , and CCR5, also showed increased expression in mdx muscle.  Ref: 12609504 Neuromuscul Disord, 2003
